# Supplementary material for: How Does Cultivar, Maturation, and Pre-Treatment Affect Nutritional, Physicochemical, and Pasting Properties of Plantain Flours?
Source: Foods. 2021 Jul 29;10(8):1749. doi: 10.3390/foods10081749 (PMC8393996; doi:10.3390/foods10081749)
Supplement: Supplementary file 1 [file foods-10-01749-s001.zip › foods-1258309-supl.pdf]

foods
MDPI

Table 1. Proximate composition of plantain flours as affected by cultivar, ripening stages, and pre-treatment methods.

Cultivars	Ripening stages[1]	Pre-treatments	Moisture[2] (g 100 g^{-1})	Crude fat (g 100 g^{-1})	Crude protein (g 100 g^{-1})	Carbohydrate (g 100 g^{-1})	Total ash (g 100 g^{-1})	Crude fiber (g 100 g^{-1})
Red Essong (French bunch)	1	Control	11.18 (0.43)	0.62 (0.07)	4.06 (0.06)	79.30 (0.41)	3.12 (0.14)	1.72 (0.02)
		Citric acid	11.39 (0.66)	0.62 (0.04)	3.95 (0.10)	79.70 (0.48)	2.43 (0.03)	1.91 (0.11)
		Blanching	10.57 (0.14)	0.48 (0.03)	3.61 (0.01)	82.09 (0.07)	1.72 (0.03)	1.53 (0.00)
	2	Control	10.97 (0.18)	0.57 (0.04)	4.44 (0.01)	79.63 (0.01)	2.76 (0.26)	1.63 (0.04)
		Citric acid	10.80 (0.20)	0.57 (0.06)	3.82 (0.02)	80.71 (0.12)	2.31 (0.11)	1.79 (0.05)
		Blanching	11.33 (0.43)	0.46 (0.01)	4.05 (0.10)	80.84 (0.29)	1.83 (0.04)	1.49 (0.02)
	3	Control	11.08 (0.03)	0.53 (0.01)	4.40 (0.01)	79.30 (0.25)	3.08 (0.16)	1.61 (0.14)
		Citric acid	10.78 (0.41)	0.56 (0.05)	4.13 (0.04)	80.42 (0.13)	2.33 (0.14)	1.78 (0.16)
		Blanching	11.47 (0.40)	0.52 (0.04)	4.05 (0.00)	80.45 (0.49)	2.12 (0.01)	1.39 (0.07)
Mbouroukou 3 (False Horn bunch)	1	Control	11.47 (0.55)	0.61 (0.11)	3.67 (0.07)	79.34 (0.51)	2.39 (0.04)	2.52 (0.09)
		Citric acid	11.50 (0.20)	0.54 (0.01)	3.67 (0.02)	79.71 (0.17)	1.98 (0.01)	2.60 (0.04)
		Blanching	11.07 (0.53)	0.55 (0.02)	3.51 (0.00)	80.70 (0.48)	1.73 (0.07)	2.44 (0.11)
	2	Control	11.77 (0.14)	0.61 (0.00)	3.61 (0.01)	79.63 (0.18)	2.18 (0.29)	2.20 (0.26)
		Citric acid	11.25 (0.11)	0.60 (0.04)	3.38 (0.07)	80.56 (0.24)	1.90 (0.09)	2.31 (0.07)
		Blanching	11.01 (0.51)	0.57 (0.02)	3.42 (0.07)	80.83 (0.39)	2.06 (0.07)	2.11 (0.10)
	3	Control	11.24 (0.01)	0.62 (0.02)	3.53 (0.04)	79.97 (0.02)	2.53 (0.08)	2.11 (0.11)
		Citric acid	11.15 (0.22)	0.56 (0.00)	3.47 (0.01)	80.69 (0.29)	2.03 (0.01)	2.14 (0.07)
		Blanching	10.78 (0.36)	0.59 (0.02)	3.43 (0.13)	81.27 (0.33)	1.83 (0.11)	2.10 (0.04)
PITA 14	1	Control	11.73 (0.12)	0.49 (0.01)	3.30 (0.10)	80.43 (0.08)	2.27 (0.04)	1.78 (0.02)
		Citric acid	11.54 (0.05)	0.53 (0.03)	3.15 (0.06)	81.10 (0.04)	1.83 (0.12)	1.85 (0.11)
		Blanching	11.22 (0.28)	0.50 (0.01)	3.08 (0.01)	81.68 (0.25)	1.72 (0.01)	1.80 (0.00)
	2	Control	11.77 (0.06)	0.63 (0.04)	3.41 (0.01)	80.38 (0.06)	2.17 (0.01)	1.64 (0.04)
		Citric acid	11.55 (0.01)	0.62 (0.01)	3.42 (0.04)	80.85 (0.00)	1.81 (0.01)	1.75 (0.01)
		Blanching	11.23 (0.01)	0.55 (0.07)	3.21 (0.15)	82.10 (0.04)	1.69 (0.10)	1.62 (0.05)
	3	Control	11.03 (0.20)	0.57 (0.05)	3.52 (0.01)	81.18 (0.19)	2.15 (0.01)	1.55 (0.02)
		Citric acid	10.92 (0.01)	0.67 (0.09)	3.52 (0.07)	81.59 (0.03)	1.71 (0.06)	1.59 (0.00)
		Blanching	11.31 (0.01)	0.55 (0.01)	3.30 (0.03)	81.62 (0.03)	1.65 (0.00)	1.57 (0.02)
PITA 27	1	Control	11.45 (0.11)	0.48 (0.03)	3.53 (0.13)	80.16 (0.40)	2.28 (0.03)	2.10 (0.10)
		Citric acid	11.95 (0.35)	0.49 (0.04)	3.29 (0.02)	80.14 (0.26)	2.02 (0.00)	2.11 (0.11)
		Blanching	11.10 (0.21)	0.45 (0.05)	2.96 (0.06)	82.17 (0.31)	1.72 (0.03)	1.60 (0.04)
	2	Control	11.24 (0.01)	0.62 (0.02)	3.83 (0.11)	80.08 (0.12)	2.24 (0.06)	1.99 (0.04)
		Citric acid	11.15 (0.22)	0.66 (0.00)	3.67 (0.00)	80.45 (0.19)	2.03 (0.01)	2.04 (0.04)
		Blanching	10.78 (0.36)	0.60 (0.01)	3.38 (0.06)	81.71 (0.25)	1.73 (0.03)	1.80 (0.04)
	3	Control	11.64 (0.06)	0.67 (0.01)	4.36 (0.14)	79.25 (0.24)	2.36 (0.09)	1.72 (0.06)
		Citric acid	10.77 (0.02)	0.61 (0.01)	4.03 (0.21)	80.74 (0.16)	2.00 (0.00)	1.85 (0.06)
		Blanching	10.80 (0.03)	0.64 (0.01)	3.66 (0.16)	81.65 (0.08)	1.70 (0.02)	1.55 (0.07)

[1]Ripening stage: 1 = mature green; 2 = green with a trace of yellow; and 3 = more green than yellow. [2]Means (± standard deviation).

Table 2. Starch composition and hydration properties of plantain flours as affected by cultivar, ripening stages, and pre-treatment methods.

**Cultivars**	**Ripening stages**[1]	**Pre-treatments**	**Amylose**[2] **(g 100 g^{-1})**	**Amylo-pectin (g 100 g^{-1})**	**TS (g 100 g^{-1})**	**RS (g 100 g^{-1})**	**AS (g 100 g^{-1})**	**WHC (g g^{-1})**	**OHC (g g^{-1})**
Red Essong (French bunch)	1	Control	30.46 (0.05)	69.54 (0.05)	82.13 (1.06)	50.23 (0.16)	31.90 (0.91)	3.18 (0.04)	2.70 (0.04)
		Citric acid	27.98 (0.00)	72.02 (0.00)	84.39 (0.27)	54.32 (0.09)	30.07 (0.36)	2.81 (0.03)	2.46 (0.01)
		Blanching	27.15 (0.11)	72.85 (0.11)	83.48 (0.26)	52.21 (0.11)	31.27 (0.38)	2.98 (0.04)	2.67 (0.05)
	2	Control	29.32 (0.16)	70.68 (0.16)	72.51 (0.27)	42.39 (0.16)	30.12 (0.42)	2.79 (0.06)	2.44 (0.04)
		Citric acid	26.89 (0.11)	73.11 (0.11)	74.70 (0.79)	46.63 (0.13)	28.07 (0.66)	2.55 (0.01)	2.26 (0.04)
		Blanching	26.21 (0.06)	73.79 (0.06)	74.41 (0.00)	43.22 (0.18)	31.20 (0.18)	2.80 (0.02)	2.40 (0.03)
	3	Control	27.91 (0.11)	72.09 (0.11)	60.36 (0.18)	33.52 (0.33)	26.84 (0.51)	2.37 (0.08)	1.95 (0.04)
		Citric acid	25.51 (0.16)	74.50 (0.16)	63.49 (0.23)	37.65 (0.23)	25.84 (0.00)	2.10 (0.03)	1.77 (0.02)
		Blanching	25.40 (0.11)	74.60 (0.11)	62.56 (0.35)	34.80 (0.33)	27.77 (0.02)	2.16 (0.01)	1.82 (0.02)
Mbouroukou 3 (False Horn bunch)	1	Control	31.18 (0.11)	68.82 (0.05)	83.22 (0.79)	53.11 (0.04)	30.11 (0.82)	3.30 (0.03)	2.78 (0.04)
		Citric acid	29.54 (0.05)	70.46 (0.00)	85.89 (0.80)	56.37 (0.16)	29.53 (0.96)	3.00 (0.03)	2.50 (0.02)
		Blanching	27.95 (0.18)	72.06 (0.11)	86.74 (0.13)	55.11 (0.01)	31.63 (0.12)	3.11 (0.02)	2.65 (0.04)
	2	Control	29.77 (0.16)	70.23 (0.00)	75.18 (0.18)	44.17 (0.13)	31.01 (0.30)	2.84 (0.08)	2.57 (0.06)
		Citric acid	28.30 (0.16)	71.71 (0.00)	78.99 (0.60)	47.77 (0.35)	31.22 (0.25)	2.61 (0.06)	2.30 (0.04)
		Blanching	27.26 (0.78)	72.74 (0.00)	77.75 (0.98)	46.26 (0.23)	31.49 (0.75)	2.81 (0.03)	2.48 (0.05)
	3	Control	28.94 (0.05)	71.06 (0.11)	61.55 (0.52)	35.36 (0.24)	26.19 (0.76)	2.44 (0.01)	2.21 (0.01)
		Citric acid	27.82 (0.00)	72.18 (0.05)	64.78 (0.66)	39.44 (0.02)	25.34 (0.64)	2.12 (0.04)	1.80 (0.03)
		Blanching	26.38 (0.11)	73.63 (0.11)	65.18 (0.22)	37.33 (0.33)	27.85 (0.54)	2.27 (0.06)	1.86 (0.02)
PITA 14	1	Control	27.38 (0.11)	72.62 (0.11)	81.64 (0.13)	49.67 (0.16)	31.97 (0.02)	2.88 (0.03)	2.36 (0.01)
		Citric acid	25.70 (0.11)	74.30 (0.11)	82.70 (0.53)	53.65 (0.51)	29.05 (0.02)	2.61 (0.04)	2.24 (0.04)
		Blanching	24.74 (0.00)	75.26 (0.00)	83.93 (0.79)	52.35 (0.13)	31.59 (0.93)	2.69 (0.03)	2.39 (0.04)
	2	Control	26.18 (0.11)	73.83 (0.11)	72.90 (0.93)	41.67 (0.48)	31.23 (0.45)	2.52 (0.09)	2.27 (0.04)
		Citric acid	23.68 (0.00)	76.32 (0.00)	74.13 (0.13)	45.15 (0.09)	28.99 (0.04)	2.31 (0.02)	2.00 (0.03)
		Blanching	23.25 (0.11)	76.76 (0.11)	72.78 (0.40)	43.34 (0.31)	29.44 (0.71)	2.41 (0.02)	2.16 (0.02)
	3	Control	25.84 (0.11)	74.17 (0.11)	63.20 (0.66)	32.72 (0.24)	30.48 (0.42)	2.16 (0.01)	1.82 (0.02)
		Citric acid	22.85 (0.16)	77.15 (0.16)	66.59 (0.01)	36.28 (0.21)	30.32 (0.19)	1.97 (0.02)	1.63 (0.03)
		Blanching	22.59 (0.11)	77.41 (0.11)	65.31 (0.18)	34.32 (0.13)	30.99 (0.05)	2.06 (0.04)	1.73 (0.01)
PITA 27	1	Control	29.77 (0.16)	70.23 (0.16)	81.54 (0.66)	49.89 (0.18)	31.65 (0.48)	2.98 (0.06)	2.48 (0.04)
		Citric acid	28.59 (0.11)	71.41 (0.11)	84.37 (1.73)	53.97 (0.26)	30.40 (0.99)	2.64 (0.02)	2.32 (0.03)
		Blanching	27.76 (0.11)	72.24 (0.11)	84.31 (0.18)	52.67 (0.49)	31.64 (0.31)	2.80 (0.02)	2.39 (0.04)
	2	Control	27.47 (0.60)	72.54 (0.60)	73.86 (0.53)	40.19 (0.06)	33.67 (0.59)	2.70 (0.04)	2.33 (0.02)
		Citric acid	26.71 (0.11)	73.29 (0.11)	75.99 (0.93)	45.75 (0.65)	30.24 (0.58)	2.41 (0.03)	2.19 (0.01)
		Blanching	25.65 (0.11)	74.36 (0.11)	74.56 (0.36)	43.59 (0.09)	30.97 (0.26)	2.50 (0.02)	2.26 (0.01)
	3	Control	24.66 (0.10)	75.34 (0.10)	61.27 (0.39)	30.91 (0.54)	30.37 (0.94)	2.28 (0.04)	1.97 (0.07)
		Citric acid	23.30 (0.17)	76.70 (0.17)	63.60 (0.40)	34.40 (0.39)	29.21 (0.78)	2.09 (0.04)	1.79 (0.01)
		Blanching	23.37 (0.49)	76.64 (0.49)	63.56 (0.79)	32.53 (0.40)	31.03 (0.40)	2.23 (0.02)	1.85 (0.01)

[1]Ripening stage: 1 = mature green; 2 = green with a trace of yellow; and 3 = more green than yellow. [2]Means (± standard deviation).

Table 3. Total phenolic, carotenoid contents, and color alterations of flours as affected by cultivar, ripening stages, and pre-treatment methods.

Cultivars	Ripening stages[1]	Pre-treatments	TP (mg GAE g^{-1})	Carotenoids (µg g^{-1})	L* (-)	a* (-)	b* (-)	C* (-)	BI (-)
Red Essong (French bunch)	1	Control	0.44 (0.01)	0.25 (0.00)	67.15 (0.16)	0.68 (0.00)	11.24 (0.00)	11.26 (0.00)	18.65 (0.05)
		Citric acid	1.60 (0.04)	1.44 (0.01)	70.33 (0.21)	0.40 (0.04)	9.46 (0.07)	9.47 (0.07)	14.53 (0.02)
		Blanching	3.07 (0.12)	3.38 (0.01)	60.12 (0.17)	0.68 (0.01)	12.44 (0.12)	12.45 (0.12)	23.48 (0.16)
	2	Control	0.30 (0.02)	0.19 (0.01)	64.54 (0.59)	0.66 (0.01)	12.35 (0.01)	12.37 (0.01)	21.51 (0.25)
		Citric acid	1.34 (0.03)	1.09 (0.04)	65.40 (0.57)	0.45 (0.01)	10.55 (0.04)	10.55 (0.04)	17.69 (0.22)
		Blanching	2.62 (0.04)	2.17 (0.06)	59.56 (0.01)	0.47 (0.01)	12.94 (0.22)	12.94 (0.22)	24.51 (0.44)
	3	Control	0.14 (0.02)	0.09 (0.05)	58.21 (0.30)	0.64 (0.05)	14.35 (0.06)	14.36 (0.06)	28.47 (0.36)
		Citric acid	1.01 (0.04)	0.96 (0.04)	61.71 (1.42)	0.46 (0.04)	12.08 (0.06)	12.08 (0.06)	21.83 (0.48)
		Blanching	2.07 (0.08)	1.85 (0.05)	57.67 (0.40)	0.32 (0.01)	13.55 (0.08)	13.55 (0.09)	26.57 (0.42)
Mbouroukou 3 (False Horn bunch)	1	Control	0.63 (0.01)	0.84 (0.03)	57.03 (0.04)	0.77 (0.01)	11.29 (0.01)	11.32 (0.01)	22.56 (0.06)
		Citric acid	1.79 (0.01)	1.65 (0.00)	60.77 (0.06)	0.54 (0.06)	9.39 (0.65)	9.41 (0.65)	17.05 (1.14)
		Blanching	3.51 (0.04)	3.78 (0.01)	55.07 (0.63)	0.82 (0.02)	12.19 (0.26)	12.21 (0.26)	25.55 (0.24)
	2	Control	0.45 (0.03)	0.65 (0.04)	56.57 (0.06)	0.73 (0.06)	12.09 (0.02)	12.11 (0.02)	24.45 (0.10)
		Citric acid	1.53 (0.01)	1.42 (0.02)	58.75 (0.04)	0.71 (0.04)	9.84 (0.08)	9.86 (0.08)	18.80 (0.12)
		Blanching	3.20 (0.02)	2.80 (0.08)	52.71 (0.07)	0.88 (0.02)	12.87 (0.02)	12.89 (0.02)	28.59 (0.13)
	3	Control	0.20 (0.02)	0.49 (0.01)	55.28 (0.03)	0.75 (0.01)	13.04 (0.04)	13.06 (0.04)	27.29 (0.09)
		Citric acid	1.16 (0.06)	1.02 (0.01)	55.88 (0.20)	0.83 (0.03)	9.93 (0.10)	9.96 (0.10)	20.23 (0.17)
		Blanching	2.78 (0.04)	2.45 (0.01)	51.49 (0.09)	0.91 (0.03)	13.44 (0.05)	13.47 (0.05)	30.87 (0.11)
PITA 14	1	Control	0.76 (0.02)	0.15 (0.01)	57.45 (0.07)	0.85 (0.01)	11.40 (0.15)	11.43 (0.15)	22.71 (0.29)
		Citric acid	1.79 (0.09)	0.79 (0.00)	58.58 (0.29)	0.75 (0.01)	9.78 (0.01)	9.81 (0.01)	18.80 (0.08)
		Blanching	4.06 (0.11)	2.82 (0.01)	56.77 (0.01)	1.04 (0.08)	11.73 (0.42)	11.78 (0.43)	24.00 (1.04)
	2	Control	0.55 (0.02)	0.11 (0.01)	56.32 (0.05)	0.85 (0.01)	11.73 (0.51)	11.76 (0.51)	23.96 (1.07)
		Citric acid	1.16 (0.08)	0.64 (0.01)	57.58 (0.29)	0.80 (0.01)	10.30 (0.01)	10.33 (0.01)	20.28 (0.11)
		Blanching	3.38 (0.05)	2.01 (0.04)	55.77 (0.01)	1.14 (0.01)	11.85 (0.02)	11.90 (0.02)	24.86 (0.04)
	3	Control	0.25 (0.06)	0.08 (0.01)	55.60 (0.16)	0.85 (0.02)	12.20 (0.04)	12.22 (0.03)	25.34 (0.03)
		Citric acid	1.01 (0.02)	0.55 (0.01)	56.46 (0.13)	0.85 (0.01)	11.29 (0.05)	11.32 (0.05)	22.91 (0.04)
		Blanching	2.96 (0.23)	1.25 (0.00)	53.39 (0.28)	1.19 (0.01)	12.30 (0.34)	12.36 (0.34)	27.27 (0.96)
PITA 27	1	Control	1.00 (0.03)	0.18 (0.01)	54.62 (0.10)	0.75 (0.00)	9.02 (0.11)	9.05 (0.11)	18.65 (0.19)
		Citric acid	1.90 (0.05)	0.95 (0.02)	57.50 (0.15)	0.69 (0.04)	8.69 (0.08)	8.72 (0.09)	16.90 (0.27)
		Blanching	3.95 (0.11)	1.47 (0.01)	49.44 (0.02)	0.78 (0.01)	9.16 (0.03)	9.19 (0.03)	21.20 (0.09)
	2	Control	0.74 (0.02)	0.12 (0.01)	53.24 (0.31)	0.76 (0.05)	9.10 (0.07)	9.13 (0.07)	19.37 (0.35)
		Citric acid	1.58 (0.05)	0.73 (0.03)	55.45 (0.42)	0.75 (0.06)	9.25 (0.24)	9.28 (0.24)	18.84 (0.75)
		Blanching	3.49 (0.05)	1.21 (0.03)	48.30 (0.06)	0.79 (0.04)	9.18 (0.06)	9.21 (0.07)	21.81 (0.20)
	3	Control	0.41 (0.02)	0.04 (0.01)	52.60 (0.28)	0.79 (0.01)	9.76 (0.04)	9.79 (0.04)	21.18 (0.05)
		Citric acid	1.20 (0.02)	0.56 (0.03)	53.35 (0.02)	0.76 (0.01)	9.66 (0.04)	9.69 (0.04)	20.58 (0.11)
		Blanching	3.04 (0.07)	1.08 (0.00)	47.10 (0.01)	0.84 (0.01)	9.32 (0.00)	9.36 (0.00)	22.89 (0.03)

[1]Ripening stage: 1 = mature green; 2 = green with a trace of yellow; and 3 = more green than yellow. [2]Means (± standard deviation).

Table 4. Pasting properties of flour as affected by cultivar, ripening stages, and pre-treatment methods.

Cultivars	Ripening stages[1]	Pre-treatments	Peak viscosity (mPa s)	Trough viscosity (mPa s)	Final viscosity (mPa s)	Breakdown (mPa s)	Setback (mPa s)	Peak time (min)	Pasting temperature (°C)
Red Essong (French bunch)	1	Control	487.2 (3.7)	309.8 (4.5)	474.5 (4.2)	177.4 (1.8)	164.7 (0.3)	5.1 (0.0)	86.5 (0.1)
		Citric acid	473.7 (3.3)	255.3 (1.6)	392.4 (9.4)	218.4 (0.8)	137.1 (0.5)	5.1 (0.0)	86.9 (0.5)
		Blanching	306.1 (2.8)	235.6 (0.5)	461.8 (0.4)	70.5 (0.5)	226.2 (1.2)	5.2 (0.0)	71.3 (0.6)
	2	Control	499.0 (0.3)	315.1 (1.9)	454.6 (3.3)	183.9 (2.3)	139.5 (3.5)	5.1 (0.1)	85.8 (0.0)
		Citric acid	485.5 (2.9)	258.2 (2.7)	368.9 (1.2)	227.3 (4.4)	110.7 (0.1)	5.1 (0.0)	86.1 (0.6)
		Blanching	313.1 (3.4)	242.9 (3.3)	437.8 (6.8)	70.2 (1.0)	194.9 (1.9)	5.2 (0.0)	70.8 (0.1)
	3	Control	516.2 (1.7)	319.4 (0.9)	443.5 (4.6)	196.8 (2.6)	124.1 (3.7)	5.0 (0.0)	84.5 (0.1)
		Citric acid	496.9 (11.6)	265.9 (6.4)	355.1 (0.4)	231.0 (5.2)	82.9 (6.0)	5.1 (0.0)	84.1 (0.7)
		Blanching	348.6 (0.5)	243.0 (17.1)	422.6 (2.5)	105.6 (0.6)	179.6 (1.6)	5.1 (0.0)	70.1 (0.0)
Mbouroukou 3 (False Horn bunch)	1	Control	440.8 (7.0)	276.6 (2.4)	465.1 (4.7)	164.2 (2.1)	188.5 (3.4)	5.3 (0.1)	86.9 (0.6)
		Citric acid	394.1 (1.1)	246.6 (2.0)	357.4 (0.1)	147.5 (9.0)	110.8 (1.9)	4.9 (0.0)	86.5 (0.0)
		Blanching	256.1 (5.4)	180.4 (13.7)	399.2 (6.4)	75.7 (2.7)	218.8 (1.4)	6.0 (0.0)	73.4 (2.3)
	2	Control	447.3 (4.1)	283.5 (2.4)	450.2 (2.7)	163.8 (3.8)	166.7 (2.1)	5.1 (0.0)	86.0 (1.1)
		Citric acid	403.5 (2.1)	251.3 (1.1)	339.8 (3.9)	152.2 (1.6)	88.5 (0.7)	4.9 (0.0)	85.9 (0.1)
		Blanching	312.0 (1.5)	205.7 (1.7)	362.9 (1.1)	106.3 (1.2)	157.2 (2.8)	5.9 (0.0)	69.0 (1.3)
	3	Control	455.0 (0.4)	295.0 (0.7)	426.7 (4.9)	160.0 (1.2)	131.7 (2.9)	5.0 (0.0)	85.0 (1.4)
		Citric acid	416.5 (3.2)	254.8 (5.0)	324.7 (3.7)	161.7 (5.3)	69.9 (0.8)	4.9 (0.0)	84.6 (0.1)
		Blanching	321.2 (3.2)	230.3 (0.8)	342.6 (3.0)	90.9 (3.9)	112.3 (2.8)	5.6 (0.1)	64.8 (1.8)
PITA 14	1	Control	468.0 (1.6)	331.8 (4.0)	497.3 (5.1)	136.2 (1.5)	165.5 (4.7)	5.1 (0.0)	89.7 (1.2)
		Citric acid	451.5 (1.8)	256.9 (3.0)	317.8 (11.6)	194.6 (4.8)	60.9 (1.0)	5.0 (0.0)	88.0 (0.0)
		Blanching	232.8 (18.9)	218.5 (13.5)	378.0 (1.7)	14.3 (0.3)	159.5 (1.1)	7.0 (0.0)	68.4 (1.2)
	2	Control	569.8 (12.6)	340.6 (0.1)	472.5 (2.9)	229.2 (1.7)	131.9 (4.2)	5.2 (0.0)	84.7 (0.9)
		Citric acid	466.5 (5.3)	266.9 (3.0)	308.0 (1.7)	199.6 (0.7)	41.1 (2.4)	4.9 (0.0)	83.7 (1.3)
		Blanching	247.8 (2.4)	228.5 (0.7)	352.8 (0.4)	19.3 (5.4)	124.3 (1.0)	6.9 (0.0)	64.7 (0.7)
	3	Control	604.2 (5.9)	363.7 (5.2)	455.9 (3.2)	240.5 (3.5)	92.2 (1.4)	5.1 (0.1)	83.5 (0.6)
		Citric acid	484.7 (2.4)	288.2 (5.8)	294.1 (6.9)	196.5 (1.1)	5.9 (0.8)	4.9 (0.1)	81.2 (0.6)
		Blanching	265.8 (1.6)	248.3 (1.5)	332.9 (13.7)	17.5 (0.1)	84.6 (0.8)	6.6 (0.4)	61.0 (0.2)
PITA 27	1	Control	560.3 (17.7)	347.1 (6.2)	570.8 (14.3)	213.2 (1.0)	223.7 (2.7)	5.0 (0.0)	88.1 (0.0)
		Citric acid	524.8 (5.8)	327.2 (4.8)	445.0 (4.4)	197.6 (1.4)	117.8 (0.5)	4.9 (0.0)	86.5 (0.0)
		Blanching	361.4 (14.3)	311.3 (0.7)	475.3 (10.6)	50.1 (3.6)	164.0 (0.6)	5.8 (0.1)	76.3 (0.3)
	2	Control	608.3 (6.7)	419.6 (1.4)	541.5 (8.2)	188.7 (8.9)	121.9 (2.5)	5.1 (0.0)	87.5 (0.5)
		Citric acid	578.1 (0.9)	365.4 (3.1)	422.8 (1.3)	212.7 (1.5)	57.4 (1.8)	4.9 (0.0)	86.9 (0.0)
		Blanching	444.5 (1.5)	335.0 (5.5)	466.1 (1.8)	109.5 (0.0)	131.1 (3.1)	5.7 (0.2)	75.8 (0.0)
	3	Control	682.7 (16.9)	473.0 (10.6)	530.3 (8.4)	209.7 (9.4)	57.3 (0.9)	5.0 (0.0)	86.0 (0.8)
		Citric acid	656.7 (10.3)	394.1 (0.5)	407.3 (3.7)	262.6 (2.8)	13.2 (1.1)	4.9 (0.0)	85.6 (0.2)
		Blanching	521.0 (7.3)	379.0 (11.9)	446.7 (4.5)	142.0 (6.7)	67.7 (3.1)	5.2 (0.1)	72.5 (2.4)

[1]Ripening stage: 1 = mature green; 2 = green with a trace of yellow; and 3 = more green than yellow. [2]Means (± standard deviation).
